# Postoperative hypotension in patients discharged to the intensive care unit after non-cardiac surgery is associated with adverse clinical outcomes

**DOI:** 10.1186/s13054-020-03412-5

**Published:** 2020-12-07

**Authors:** Nathan J. Smischney, Andrew D. Shaw, Wolf H. Stapelfeldt, Isabel J. Boero, Qinyu Chen, Mitali Stevens, Ashish K. Khanna

**Affiliations:** 1 Department of Anesthesiology and Critical Care Medicine, Mayo Clinic, 200 First St SW, Rochester, MN 55905 USA; 2 Department of Critical Care Medicine, University of Alberta, Edmonton, Canada; 3 Department of Anesthesiology and Pain Medicine, University of Alberta, Edmonton, Canada; 4 Department of Anesthesiology and Critical Care Medicine, Saint Louis University, St. Louis, MO USA; 5 Boston Consulting Group, Boston, MA USA; 6 Edwards Lifesciences, Irvine, CA USA; 7 Department of Anesthesiology, Section on Critical Care Medicine, Wake Forest University School of Medicine, Wake Forest Baptist Health, Winston-Salem, NC USA; 8 Outcomes Research Consortium, Cleveland, OH USA

**Keywords:** Acute kidney injury (AKI), All-cause mortality, Critically ill patients, Dialysis, Intensive care setting, Major adverse cardiac or cerebrovascular events (MACCE), Mean arterial pressure, 90-day mortality, Postoperative hypotension, 30-day mortality

## Abstract

**Background:**

The postoperative period is critical for a patient’s recovery, and postoperative hypotension, specifically, is associated with adverse clinical outcomes and significant harm to the patient. However, little is known about the association between postoperative hypotension in patients in the intensive care unit (ICU) after non-cardiac surgery, and morbidity and mortality, specifically among patients who did not experience intraoperative hypotension. The goal of this study was to assess the impact of postoperative hypotension at various absolute hemodynamic thresholds (≤ 75, ≤ 65 and ≤ 55 mmHg), in the absence of intraoperative hypotension (≤ 65 mmHg), on outcomes among patients in the ICU following non-cardiac surgery.

**Methods:**

This multi-center retrospective cohort study included specific patient procedures from Optum® healthcare database for patients without intraoperative hypotension (MAP ≤ 65 mmHg) discharged to the ICU for ≥ 48 h after non-cardiac surgery with valid mean arterial pressure (MAP) readings. A total of 3185 procedures were included in the final cohort, and the association between postoperative hypotension and the primary outcome, 30-day major adverse cardiac or cerebrovascular events, was assessed. Secondary outcomes examined included all-cause 30- and 90-day mortality, 30-day acute myocardial infarction, 30-day acute ischemic stroke, 7-day acute kidney injury stage II/III and 7-day continuous renal replacement therapy/dialysis.

**Results:**

Postoperative hypotension in the ICU was associated with an increased risk of 30-day major adverse cardiac or cerebrovascular events at MAP ≤ 65 mmHg (hazard ratio [HR] 1.52; 98.4% confidence interval [CI] 1.17–1.96) and ≤ 55 mmHg (HR 2.02, 98.4% CI 1.50–2.72). Mean arterial pressures of ≤ 65 mmHg and ≤ 55 mmHg were also associated with higher 30-day mortality (MAP ≤ 65 mmHg, [HR 1.56, 98.4% CI 1.22–2.00]; MAP ≤ 55 mmHg, [HR 1.97, 98.4% CI 1.48–2.60]) and 90-day mortality (MAP ≤ 65 mmHg, [HR 1.49, 98.4% CI 1.20–1.87]; MAP ≤ 55 mmHg, [HR 1.78, 98.4% CI 1.38–2.31]). Furthermore, we found an association between postoperative hypotension with MAP ≤ 55 mmHg and acute kidney injury stage II/III (HR 1.68, 98.4% CI 1.02–2.77). No associations were seen between postoperative hypotension and 30-day readmissions, 30-day acute myocardial infarction, 30-day acute ischemic stroke and 7-day continuous renal replacement therapy/dialysis for any MAP threshold.

**Conclusions:**

Postoperative hypotension in critical care patients with MAP ≤ 65 mmHg is associated with adverse events even without experiencing intraoperative hypotension.

## Background

The postoperative period can be extremely dangerous for patients, and postoperative hypotension (POH), specifically, is associated with significant harm [1]. Mortality is 1000 times more common postoperatively than intraoperatively [2], and if mortality within 30 days of surgery was classified as a separate indication, it would be the third leading cause of death in the USA [3]. Myocardial infarction is the leading cause of attributable postoperative death [4], and other common pathologies include acute ischemic stroke (AIS) and acute kidney disease [3]. Furthermore, escalation of the complexity of postoperative critical care is not only costly, but is also associated with a decreased quality of life in the years following hospitalization [5].

Despite its association with significant patient harm and cost, only a limited number of studies have investigated POH, particularly none previously in cohorts without preceding intraoperative hypotension (IOH) [1, 6]. A mixed cohort of critically ill patients (including patients from the coronary care unit, general, medical, cardiac and surgical intensive care units (ICU)) revealed an increased risk of myocardial injury, mortality and kidney injury at MAP thresholds of 85 mmHg with a progressive increase at lower pressures [7]. A specific investigation in a postoperative population demonstrated that patients in the surgical ICU following non-cardiac surgery are especially sensitive to even mild amounts of hypotension [8]. However, taking a closer look at the data, post hoc analyses revealed that the relationship between ICU hypotension and the risk of adverse outcomes was dependent on the amount of IOH [8]. Moreover, there is a paucity of literature which examines various absolute blood pressure thresholds in postoperative ICU patients.

Understanding the impact of POH at different hemodynamic thresholds without the contribution from IOH on adverse clinical outcomes among post-surgical critical care patients can provide better insight into the importance of POH management and potentially inform strategies to assist in early intervention. For example, in a recent randomized clinical trial (HYPE), the use of a machine learning-derived early warning system resulted in less IOH and potentially fewer adverse outcomes (although not statistically significant) than the standard of care [9]. Furthermore, continuous hemodynamic monitoring and individualized blood pressure management might assist early intervention and thus result in overall better clinical outcomes, including risk reduction for postoperative organ dysfunction such as risk of AKI [10, 11].

This multi-center retrospective cohort study sought to evaluate the association of POH across multiple hemodynamic thresholds (≤ 75, ≤ 65 and ≤ 55 mmHg), in the absence of IOH (≤ 65 mmHg), among patients in the ICU after non-cardiac surgery. The primary outcome was major adverse cardiac or cerebrovascular events (MACCE) in the first 30 days. Additional clinical adverse outcomes investigated included: all-cause 30- and 90-day mortality, 30-day AMI, 30-day AIS, 7-day AKI stage II/III, 30-day readmissions and the need for continuous renal replacement therapy (CRRT)/dialysis in the first 7 days.

## Materials and methods

### Data source

The cohort for this study was obtained from the Optum® healthcare database (Optum®, Eden Prairie, MN), which integrates and standardizes de-identified electronic medical records from both ambulatory and inpatient settings of over 2000 hospitals and 7000 clinics. Optum® data cover diagnoses and procedure codes, clinical observations (i.e., vital signs), medications and laboratory tests [12]. In advance, a statistical analysis plan was submitted to the Western Institutional Review Board (Puyallup, WA), and the study was determined exempt from review as it does not meet the definition of human research as defined in 45 Code of Federal Regulations 46.102.

### Cohort selection

Patients were identified from an original cohort of 368,222 non-cardiac/non-obstetric surgical procedures (January 1, 2008, to December 31, 2017; based upon data availability) with valid intraoperative MAP readings (see Exposure section for calculations) and at least 1 year each of pre-surgical history and of follow-up (Additional file 1: Fig. S1, for initial attrition). No patient age restriction was applied.

The final cohort of 3185 non-cardiac/non-obstetric procedures for patients discharged to the ICU (with length of stay ≥ 48 h) with no evidence of IOH (MAP ≤ 65 mmHg) was selected by applying the following exclusion criteria: (a) patient died within 24 h of surgery; (b) procedures with greater than two 5-h gaps between MAP readings [13] within 48 h post-surgery; (c) missing or invalid MAP values within 48 h post-surgery; (d) discharge setting listed as the medical/surgical ward; (e) multiple conflicting discharge locations (“invalid”); (f) patients discharged to the ICU with greater than one 2-h interval between MAP readings over the 48 h post-surgery (this more stringent requirement for MAP readings [versus step (b)] was applied due to the ICU setting); and (g) IOH exposure (MAP ≤ 65 mmHg based on the literature [14, 15]) during qualifying surgery.

The surgeries were identified using procedures in the Center for Disease Control’s National Healthcare Safety Network Surgical Site Infection monitoring program, and the International Classification of Disease (ICD)-9 and 10, and Healthcare Common Procedure Coding System codes [16, 17]. For patients with multiple procedures within 30 days of each other, the last surgery was used as the index procedure. All procedures were included in cases where surgeries were > 30 days apart.

A machine learning approach was utilized to identify patients discharged to the ICU for patients with an undocumented post-surgery care location as previously described [18] (see Additional file 1: Method S1, for brief description of the machine learning approach). From the final cohort of 3185 patients, 46.7% of patients (*n* = 1486) had a formal documented discharge to the ICU, and 53.3% of patients (*n* = 1699) were identified as discharged to the ICU using the algorithm.

### Determining MAP thresholds and exposures for postoperative hypotension

**Fig. 1:**
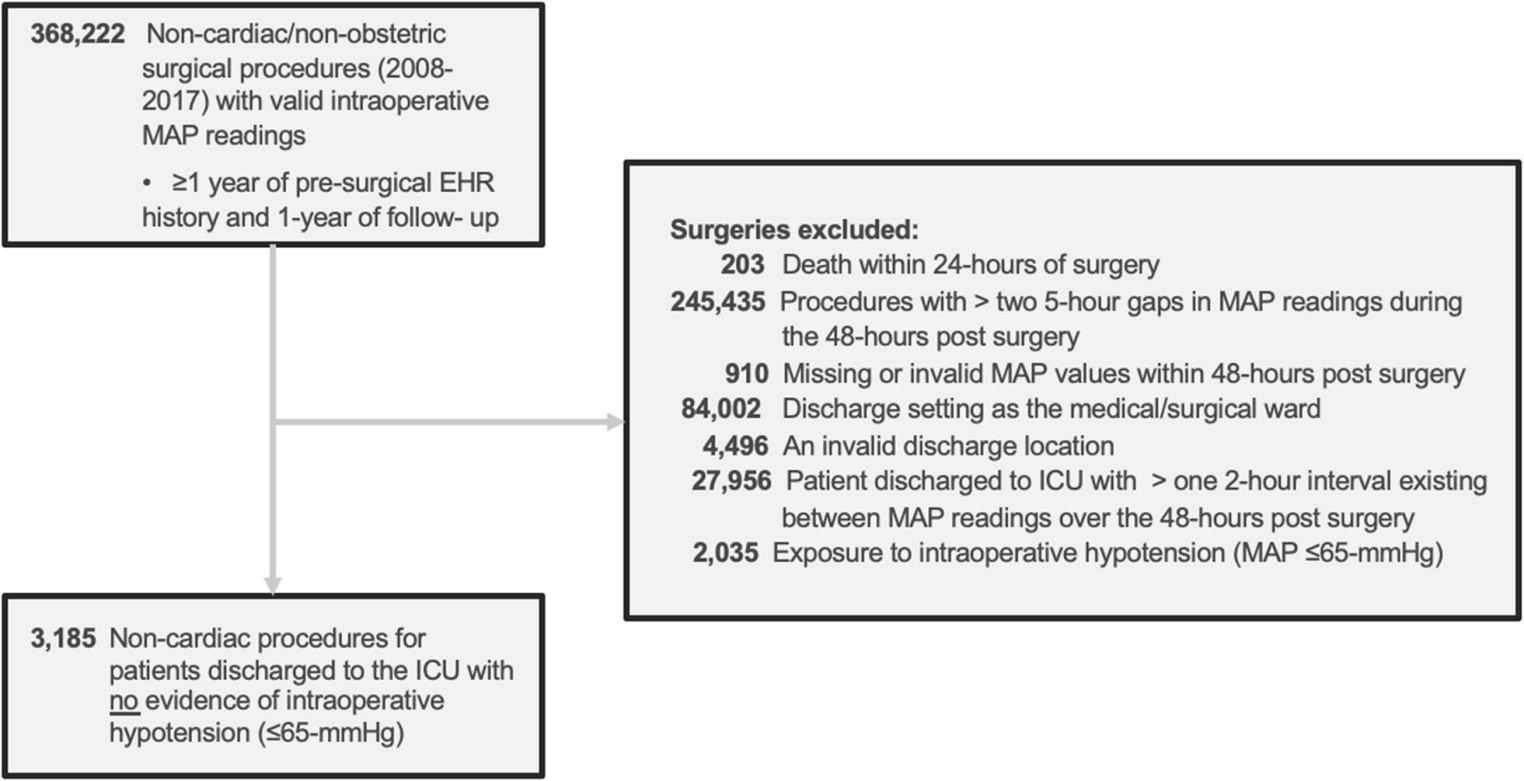
Patient cohort attrition diagram. The final study cohort comprised 3185 procedures of 3169 unique patients. MAP, mean arterial pressure

**Fig. 2:**
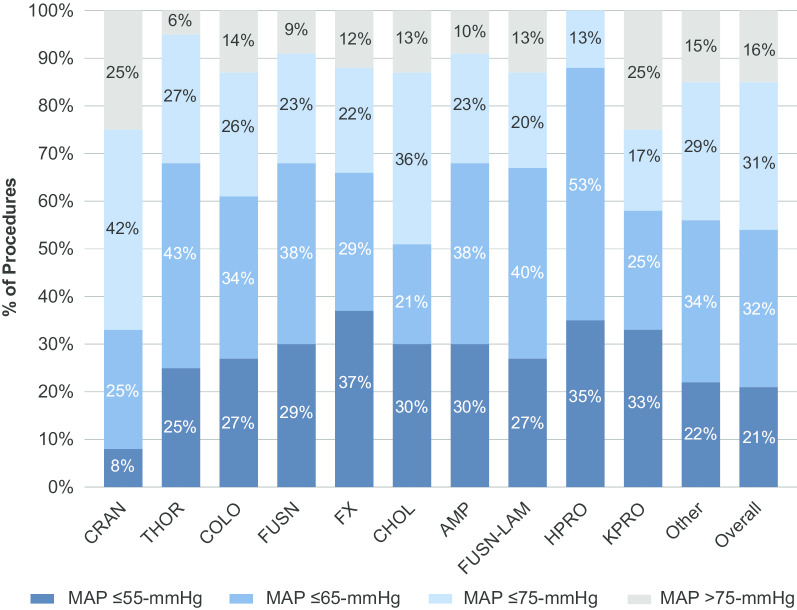
Cumulative incidence of postoperative hypotension for overall surgeries and the top 10 surgeries, among patients discharged to the ICU for 48 h after non-cardiac/non-obstetric surgery. Patients included had no preceding IOH (MAP ≤ 65 mmHg). The incidence of lowest POH value recorded per patient by MAP thresholds of ≤ 55 mmHg, ≤ 65 mmHg, ≤ 75 mmHg and > 75 mmHg is shown for overall surgeries and the 10 most common surgery types and overall (craniotomy most common, knee prosthesis least common). Due to rounding, categories will not always add to 100%. surgeries in the top 10 cohort: AMP, limb amputation; CHOL, gallbladder surgery; COLO, colon surgery; CRAN, craniotomy; FUSN, spinal fusion; FUSN-LAM, spinal fusion laminectomy; FX, open reduction of fracture; HPRO, hip prosthesis; KPRO, knee prosthesis; THOR, thoracic surgery (non-cardiac, non-vascular); POH, postoperative hypotension

MAP was calculated by using the following formula: MAP = [(2 × diastolic blood pressure) + systolic blood pressure]/3. Invalid MAP data were identified using criteria described in previously published sources [14]. 0.7% (*n* = 844) of patient procedures were excluded due to missing or invalid MAP readings within the 48 h post-surgery (Fig. 1). Based on available literature, three a priori defined absolute MAP thresholds (≤ 75, ≤ 65 and ≤ 55 mmHg), were used [15]. POH was assessed as a binary variable (presence/absence; POH for the relevant threshold was defined as a single MAP reading below the threshold) with the defined absolute MAP thresholds over the first 48 h post-surgery (beginning from surgical stop time). Additionally, we accounted for the timing of the binary POH exposure to ensure that it occurred before any outcome of interest. We also report the incidence of the hypotension exposure by surgery type (Fig. 2).

### Potential confounding variables

Potential confounding variables were defined a priori based on previously described methods [13] and on clinical and operative factors that may affect the odds of experiencing MACCE (Tables 1 and 2; Additional file 1: Table S1). Briefly, patient demographics were determined, and comorbidities were identified in the year before surgery using ICD-9/10 codes except for valvular disease and severity, which were identified using physician notes (see Additional file 1: Table S2, for ICD codes). The Charlson comorbidity index (CCI) was utilized to determine baseline patient comorbidity severity [19]. Additional procedures in the year prior to surgery, besides the index procedure, were captured from relevant ICD-9/10 and Current Procedural Terminology (CPT) codes. The use of antihypertensive medication in the year before the procedure was captured from patient medication records (see Additional file 1: Table S3, for a list of antihypertensive drugs). Vasopressor agents were collected from patient records; evidence of major bleeding (ICD-9/10) and use of antihypertensives within 48 h after surgery were included as time-dependent confounding variables.

**Table 1:** Cohort baseline characteristics

Patient characteristics	POH MAP threshold				
	Overall (*n* = 3185)	≤ 55 mmHg (*n* = 654)	≤ 65 mmHg (*n* = 1688)	≤ 75 mmHg (*n* = 2674)	> 75 mmHg (*n* = 511)
Gender					
Male	1658 (52.1%)	289 (44.2%)	848 (52.0%)	1339 (49.9%)	192 (37.6%)
Female	1527 (47.9%)	365 (55.8%)	649 (48.0%)	1335 (50.1%)	319 (62.4%)
Race					
Caucasian	2462 (77.3%)	541 (82.7%)	1371 (81.2%)	2105 (78.7%)	357 (69.9%)
African-American	411 (12.9%)	57 (8.7%)	159 (9.4%)	310 (11.6%)	101 (19.8%)
Asian	37 (1.2%)	4 (0.6%)	18 (1.1%)	31 (1.2%)	6 (1.2%)
Other/Unknown	275 (8.6%)	52 (8.0%)	140 (8.3%)	228 (8.5%)	47 (9.2%)
Age (years), mean (± SD)	63.5 (16.4)	67.2 (15.9)	65.8 (15.9)	64.4 (16.3)	59.0 (15.8)
Region					
Midwest	1400 (44.0%)	279 (42.7%)	746 (44.2%)	1198 (44.8%)	202 (39.5%)
North	52 (1.6%)	6 (0.9%)	25 (1.5%)	49 (1.8%)	3 (0.6%)
South	1446 (45.4%)	307 (46.9%)	771 (45.7%)	1189 (44.5%)	257 (50.3%)
West	194 (6.1%)	49 (7.5%)	110 (6.5%)	173 (6.5%)	21 (4.1%)
Other/unknown	93 (2.9%)	13 (2.0%)	36 (2.1%)	65 (2.4%)	28 (5.5%)
Income					
Quartile 1	1006 (31.6%)	226 (34.6%)	570 (33.8%)	839 (31.4%)	167 (32.7%)
Quartile 2	1155 (36.3%)	234 (35.8%)	591 (35.0%)	978 (36.6%)	177 (34.6%)
Quartile 3	652 (20.5%)	121 (18.5%)	329 (19.5%)	553 (20.7%)	99 (19.4%)
Quartile 4	286 (9.0%)	59 (9.0%)	160 (9.5%)	240 (9.0%)	46 (9.0%)
Missing value	86 (2.7%)	14 (2.1%)	38 (2.3%)	64 (2.4%)	22 (4.3%)
Within 30 days before surgery					
Acute myocardial infarction^*^	172 (5.4%)	52 (8.0%)	105 (6.2%)	155 (5.8%)	17 (3.3%)
Acute ischemic stroke^*^	340 (10.7%)	44 (6.7%)	148 (8.8%)	267 (10.0%)	73 (14.3%)
Acute kidney injury	916 (28.8%)	259 (39.6%)	574 (34.0%)	794 (29.7%)	122 (23.9%)
Dialysis	113 (3.6%)	34 (5.2%)	80 (4.7%)	100 (3.7%)	13 (2.5%)
Continuous renal replacement therapy^*^	92 (2.9%)	28 (4.3%)	64 (3.8%)	81 (3.0%)	11 (2.2%)
Within 7 days before surgery					
Delirium	283 (8.9%)	44 (6.7%)	134 (7.9%)	233 (8.7%)	50 (9.8%)
Electrolyte disorder	1677 (52.7%)	381 (58.3%)	934 (55.3%)	1421 (53.1%)	256 (50.1%)
Sepsis	695 (21.8%)	192 (29.4%)	441 (26.1%)	615 (23.0%)	80 (15.7%)
Admitted from					
Home	1636 (51.4%)	345 (52.8%)	864 (51.2%)	1376 (51.5%)	260 (50.9%)
Inpatient	1315 (41.3%)	267 (40.8%)	697 (41.3%)	1096 (41.0%)	219 (42.9%)
Skilled nursing facility	183 (5.8%)	34 (5.2%)	99 (5.9%)	157 (5.9%)	26 (5.1%)
Unknown	51 (1.6%)	8 (1.2%)	28 (1.7%)	45 (1.7%)	6 (1.2%)
Procedures in the year before date of surgery					
Dialysis^*^	139 (4.4%)	46 (7.0%)	97 (5.8%)	121 (4.5%)	18 (3.5%)
Coronary artery bypass graft	30 (0.9%)	6 (0.9%)	19 (1.1%)	29 (1.1%)	1 (0.2%)
Percutaneous coronary intervention	52 (1.6%)	14 (2.1%)	34 (2.0%)	45 (1.7%)	7 (1.4%)
Use of antihypertensives	2874 (90.2%)	578 (88.4%)	1504 (89.1%)	2392 (89.5%)	482 (94.3%)

General Demographic Information and Patient Histories for non-cardiac surgery patients discharged to the ICU after surgery without intraoperative hypotension (MAP ≤ 65 mmHg)

Due to rounding, categories will not always add to 100%. *These variables were not used for model adjustment but were used to assess patient status and determine new onset of outcomes. POH, postoperative hypotension; MAP, mean arterial pressure; SD, standard deviation

**Table 2:** Cohort baseline characteristics for comorbidities and Charlson comorbidity index

Patient characteristics	POH MAP threshold				
	Overall (*n* = 3185)	≤ 55 mmHg (*n* = 654)	≤ 65 mmHg (*n* = 1688)	≤ 75 mmHg (*n* = 2674)	> 75 mmHg (*n* = 511)
Comorbidities					
Myocardial infarction	471 (14.8%)	115 (17.6%)	272 (16.1%)	408 (15.3%)	63 (12.3%)
Cerebrovascular accident	1075 (33.8%)	155 (23.7%)	471 (27.9%)	860 (32.2%)	215 (42.1%)
COPD	1157 (36.3%)	284 (43.4%)	657 (38.9%)	1001 (37.4%)	156 (30.5%)
Heart failure	699 (22.0%)	197 (30.1%)	444 (26.3%)	611 (22.9%)	88 (17.2%)
Pulmonary circulatory disorder	243 (7.6%)	73 (11.2%)	158 (9.4%)	221 (8.3%)	22 (4.3%)
Peripheral vascular disease	656 (20.6%)	171 (26.2%)	395 (23.4%)	578 (21.6%)	78 (15.3%)
Hypertension	2519 (76.0%)	488 (74.6%)	1270 (75.2%)	2012 (75.2%)	407 (79.7%)
Paralysis	392 (12.3%)	42 (6.4%)	162 (9.60%)	314 (11.7%)	78 (15.3%)
Diabetes	1099 (34.5%)	224 (34.3%)	581 (34.4%)	927 (34.7%)	172 (33.7%)
Hypothyroidism	574 (18.0%)	149 (22.8%)	355 (21.0%)	502 (18.8%)	72 (14.1%)
Renal disease	1170 (36.7%)	286 (43.7%)	681 (40.3%)	1005 (37.6%)	165 (32.3%)
Liver disease	421 (13.2%)	100 (15%)	244 (14%)	362 (14%)	59 (12%)
Lymphoma	42 (1.3%)	12 (1.8%)	21 (1.2%)	34 (1.3%)	8 (1.6%)
Solid tumor (local)	949 (29.8%)	207 (31.7%)	524 (31.0%)	825 (30.9%)	124 (24.3%)
RA/ collagen vascular disease	148 (4.7%)	33 (5.1%)	78 (4.6%)	126 (4.7%)	22 (4.3%)
Coagulopathy	259 (8.1%)	53 (8.1%)	149 (8.8%)	228 (8.5%)	31 (6.1%)
Obesity	714 (22.4%)	148 (22.6%)	363 (21.5%)	600 (22.4%)	114 (22.3%)
Anemia	1801 (56.6%)	436 (66.7%)	1045 (61.9%)	1564 (58.5%)	237 (46.4%)
Alcohol abuse	261 (8.2%)	49 (7.5%)	129 (7.6%)	209 (7.8%)	52 (10.2%)
Drug abuse	249 (7.8%)	43 (6.6%)	103 (6.1%)	198 (7.4%)	51 (10.0%)
Smoking	877 (27.5%)	167 (25.5%)	454 (26.9%)	728 (27.2%)	149 (29.2%)
Depression	784 (24.6%)	173 (26.5%)	410 (24.3%)	661 (24.7%)	123 (24.1%)
Sleep apnea	502 (15.8%)	103 (15.8%)	262 (15.5%)	412 (15.4%)	90 (17.6%)
Dementia	154 (4.8%)	36 (5.5%)	93 (5.5%)	131 (4.9%)	23 (4.5%)
Chronic kidney disease^*^	775 (24.3%)	204 (31.2%)	471 (27.9%)	674 (25.2%)	101 (19.8%)
Home oxygen	178 (5.6%)	44 (6.7%)	112 (6.6%)	161 (6.0%)	17 (3.3%)
Right-side valve disease	71 (2.2%)	23 (3.5%)	47 (2.8%)	66 (2.5%)	5 (1.0%)
Left-side valve disease	135 (4.2%)	41 (6.3%)	92 (5.5%)	122 (4.6%)	13 (2.5%)
Charlson comorbidity index (CCI)					
0	493 (15.5%)	94 (14.4%)	241 (14.3%)	409 (15.3%)	84 (16.4%)
1	591 (18.6%)	91 (13.9%)	278 (16.5%)	473 (17.7%)	118 (23.1%)
2	519 (16.3%)	112 (17.1%)	284 (16.8%)	439 (16.4%)	80 (15.7%)
3	435 (13.7%)	86 (13.2%)	244 (14.5%)	372 (13.9%)	63 (12.3%)
≥ 4	1147 (36.0%)	271 (41.4%)	641 (38.0%)	981 (36.7%)	166 (32.5%)

Comorbidities and Charlson comorbidity index (CCI) for non-cardiac surgery patients discharged to the ICU after surgery without intraoperative hypotension (MAP ≤ 65 mmHg)

Due to rounding, categories will not always add to 100%. *This comorbidity was not used for model adjustment but was used to assess patient status and determine new onset of outcomes. POH, postoperative hypotension, MAP, mean arterial pressure; RA rheumatoid arthritis, COPD, Chronic Obstructive Pulmonary Disease

In order to determine new onset of outcomes and to adjust for patient status immediately prior to the index procedure, the following conditions were identified in the 7 days prior to index procedure using the appropriate ICD-9/10 codes: delirium, electrolyte disorders, AMI, AIS and new-onset AKI. Pre-existing dialysis or CRRT was identified for a patient over the year prior to the index procedure date.

Total surgery time was estimated using the high-frequency MAP datapoints defined as intervals of ≤ 5 min between readings. Time of surgery (day/night—defined as after 6PM/6AM local time) and date (weekday/weekend) were included in the association prediction models to adjust for the association between surgical/facility factors and the likelihood of an adverse event.

### Primary, secondary and exploratory outcomes

The primary outcome was 30-day MACCE as defined by the composite measure of all-cause mortality, AMI or AIS [20–22]. Mortality was captured using the Social Security Index, and AMI and AIS were identified from ICD-9/10 codes. Additionally, AMI was captured using the Clinical Classifications Software diagnosis code 100.

Secondary outcomes investigated included: all-cause 30- and 90-day mortality, 30-day AMI, 30-day AIS, 7-day AKI stage II or III [23], 30-day readmissions and 7-day CRRT/dialysis. AKI stage II/III was chosen based on previous literature showing a significant increase in hospital mortality with stage II and III renal dysfunction [24] and defined as postoperative creatinine two times greater than the most recent preoperative value, an increase in serum creatinine ≥ 4 mg/dL, or initiation of dialysis therapy [25]. A new hospital admission within 30 days post-discharge from index hospitalization was counted within 30-day readmissions. The frequency of new-onset CRRT or intermittent hemodialysis, and delirium were assessed using ICD-9/10 codes or CPT codes.

Exploratory outcomes included readmission to ICU after 72 h post-ICU discharge, ICU-free days in the first 30 days (if a patient died in the hospital this variable counted as zero) and days alive and free (DAF) of vasopressors (calculated by subtracting days on vasopressors from the lesser of 28 or the number of days until death, as previously described) [26]. Readmission to ICU after 72 h was defined as ICU admission after 72 h after discharge from the ICU. Outcomes investigated in this study were not limited to events in the index visit.

### Statistical analysis

The associations between POH thresholds and outcomes were evaluated using two-tailed hypothesis testing. The rate of adverse events for each POH threshold was evaluated, where the reference group was defined as all patients who did not experience POH at any MAP threshold (i.e., for ≤ 65 mmHg, this is > 65 mmHg). Additionally, the mean (SD) and median (25^th^ and 75^th^ percentile) of time below MAP < 55 mmHg were calculated. Patients with an outcome (within 7 days for AIS, AMI, AKI or delirium or within 1-year for dialysis or CRRT) in the 30 days before surgery were excluded from the corresponding outcome analyses.

The independent association of POH exposures on the primary and secondary outcomes was calculated using Cox proportional hazards models, where POH was modeled as a time-dependent covariate. For secondary outcomes that did not include mortality or a length-of-stay component, Fine–Gray regression models [27], which account for the competing risk of death, were performed, and sub-distribution hazards are reported. DAF of vasopressors was assessed via Poisson regression. ICU-free days in the first 30 days were assessed via Poisson regression as it broke the linearity assumption.

A sensitivity analysis for primary and secondary outcomes was performed to evaluate the magnitude of an unobserved or unaccounted confounding effect by calculating the E-values, defined as the effect required to reduce the observed odds ratio for an outcome to 1.0 [28].

Because outcomes were evaluated across three POH thresholds, a Bonferroni correction [29] with a *p* value of ≤ 0.05/3 or 0.016 was used. ICU-free days and DAF of vasopressors were reported as incidence rate ratios (IRRs) with 98.4% confidence intervals (CIs); all other outcomes were reported as hazard ratios (HRs) with 98.4% (CIs). All analyses were conducted using SAS version 9.4 (SAS Institute Inc, Cary, NC) and R 3.5.2.

### Sample size considerations

A previous study in the USA (2004–2013) found that MACCE occurs in 3% of 10,581,621 hospitalizations for major non-cardiac surgery [20]. We would hypothesize that a 3% difference in the rate of MACCE would translate to an expected sample size of 999 with 95% confidence interval and 90% power; therefore, our study with 3185 procedures should be well above this threshold.

## Results

### Study cohort and patient characteristics

The final study cohort comprised 3185 procedures of patients (3169 unique patients) who were admitted to the ICU after non-cardiac/non-obstetric surgery with no evidence of IOH (MAP ≤ 65 mmHg) (Fig. 1). Among these patients, 47.9% were female (*n* = 1527), 77.3% were Caucasian (*n* = 2462), 36.0% (*n* = 1147) were classified with CCI category ≥ 4 and their mean (± SD) age was 63.5 (± 16.4) years (Tables 1 and 2). Patients ≤ 18 years comprised 1.2% (*n* = 37) of the population. Of the cohort, 2674 (84.0%) experienced a MAP ≤ 75 mmHg in the ICU; 1688 (53.0%) experienced hypotension with a MAP ≤ 65 mmHg and 654 (20.5%) a MAP ≤ 55 mmHg. Analysis of MAP readings suggested that ICU patients spent an average (SD) of 64.0 (123.6) minutes with MAP ≤ 55 mmHg [median (25th, 75th): 25.0 (7.9, 70.1) mins], an average of 250.6 (336.1) minutes with MAP ≤ 65 mmHg [median (25th, 75th): 108.9 (30.3, 332.2) and an average of 733.8 (716.8) minutes with MAP ≤ 75 mmHg [median (25th, 75th): 477.2 (135.3, 1161.8) mins]. Examination of the degree of hypotension experienced by patients who underwent the top 10 non-cardiac surgeries revealed that patients undergoing craniotomy procedures appear to experience less hypotension than other procedures (Fig. 2).

The overall incidence of 30-day MACCE was 17.1%. Stratified by MAP threshold, the incidence of MACCE among ICU patients without IOH was: 404 (17.7%) with MAP ≤ 75 mmHg and 293 (20.2%) and 142 (25.2%) with MAP ≤ 65 mmHg and ≤ 55 mmHg, respectively (Table 3). Descriptive statistics for patient characteristics and confounding variables stratified by POH MAP exposure thresholds, in nested groups, are shown in Tables 1 and 2 as well as Additional file 1: Table S1.

**Table 3:** Rate of adverse events among non-cardiac surgery patients discharged to the intensive care unit

Adverse outcome	POH MAP threshold				
	Overall (*n* = 3185)	≤ 55 mmHg (*n* = 654)	≤ 65 mmHg (*n* = 1688)	≤ 75 mmHg (*n* = 2674)	> 75 mmHg (*n* = 511)
**30-day MACCE**					
Total number of patients	2710	564	1452	2283	427
Patients with event (%)	464 (17.1%)	142 (25.2%)	293 (20.2%)	404 (17.7%)	60 (14.1%)
**30-day mortality**					
Total number of patients	3185	654	1688	2674	511
Patients with event (%)	519 (16.3%)	172 (26.3%)	340 (20.1%)	454 (17.0%)	65 (12.7%)
**90-day mortality**					
Total number of patients	3185	654	1688	2674	511
Patients with event (%)	642 (20.2%)	204 (31.2%)	415 (24.6%)	560 (20.9%)	82 (16.1%)
**30-day AIS**					
Total number of patients	2845	610	1540	2402	438
Patients with event (%)	75 (2.6%)	11 (1.8%)	35 (2.3%)	58 (2.4%)	17 (3.9%)
**7-day AKI stage II/III**					
Total number of patients	2269	395	1114	1880	389
Patients with event (%)	214 (9.4%)	60 (15.2%)	130 (11.7%)	194 (10.3%)	20 (5.1%)
**30-day AMI**					
Total number of patients	3013	602	1583	2519	494
Patients with event (%)	37 (1.2%)	8 (1.3%)	24 (1.5%)	33 (1.3%)	4 (0.8%)
**30-day readmission**					
Total number of patients	2702	485	1375	2258	444
Patients with event (%)	505 (18.7%)	92 (19.0%)	271 (19.7%)	430 (19.0%)	75 (16.9%)
**7-day CRRT/dialysis**					
Total number of patients	3046	608	1591	2553	493
Patients with event (%)	42 (1.4%)	20 (3.3%)	30 (1.9%)	39 (1.5%)	3 (0.6%)

ICU patients were without intraoperative hypotension (MAP < 65 mmHg) and are stratified by postoperative MAP level (≤ 75, ≤ 65 and ≤ 55 mmHg). Primary and secondary outcomes are shown.

AIS, acute ischemic stroke; AKI, acute kidney injury; AMI, acute myocardial infarction; MACCE, major adverse cardiac and cerebrovascular events; MAP, mean arterial pressure. CRRT, continuous renal replacement therapy. AMI and AIS were captured using ICD 9/10 codes (see Additional file 1: Table S2, for ICD codes). AKI stage II/III was defined using the following definition: postoperative creatinine two times greater than the most recent preoperative value, an increase in serum creatinine ≥ 4 mg/dL, or initiation of dialysis therapy

### Association between POH and MACCE (with absence of IOH) in ICU setting

**Fig. 3:**
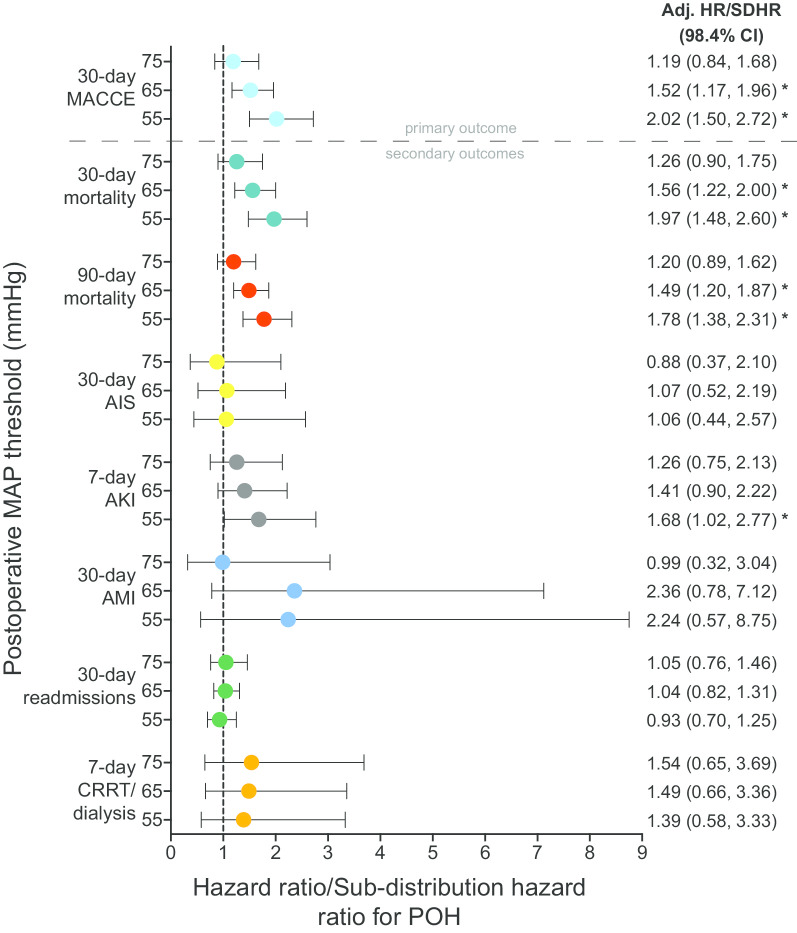
Adjusted hazard and sub-distribution hazard ratios for critical care patients with postoperative hypotension. Data shown for procedures (*n* = 3185) without preceding IOH (≤ 65 mmHg) at three absolute POH thresholds (≤ 55, ≤ 65 and ≤ 75 mmHg). *Significant after applying Bonferroni adjustment (*p* value of ≤ 0.05/3 or 0.016). POH, postoperative hypotension; MAP, mean arterial pressure; MACCE, major adverse cardiovascular or cerebrovascular events; AIS, acute ischemic stroke; AKI, acute kidney injury; AMI, acute myocardial infarction, Adj, adjusted; CRRT, continuous renal replacement therapy; HR, hazard ratio; SDHR, sub-distribution hazard ratio; CI, confidence interval

Adjusted models for the hazard ratios for ICU patients with POH without preceding IOH revealed the risk of 30-day MACCE progressively increased as the severity of hypotension increased. Both POH of MAP ≤ 65 mmHg (HR 1.52, 98.4% CI 1.17–1.96; *p* < 0.001) and MAP ≤ 55 mmHg (HR 2.02, 98.4% CI 1.50–2.72; *p* < 0.001) were associated with increased risk of MACCE. However, the association between MAP ≤ 75 mmHg (HR 1.19, 98.4% CI 0.84–1.68; *p* = 0.224) was not significant (Fig. 3 and Additional file 1: Table S4, for *p* values). Univariate (unadjusted) hazard ratios (HR) for MACCE were as follows for MAP thresholds: MAP ≤ 75 mmHg (HR 1.41, 98.4% CI 1.02–1.95; *p* < 0.011), MAP ≤ 65 mmHg (HR 1.64, 98.4% CI 1.29–2.08; *p* < 0.001), MAP ≤ 55 mmHg (HR 1.77, 98.4% CI 1.37–2.29; *p* < 0.001). Multivariate model results are included in Additional file 1: Table S4.

### POH in the ICU and associations with secondary endpoints

POH with MAP thresholds ≤ 65 mmHg and ≤ 55 mmHg was associated with higher 30-day (MAP ≤ 65 mmHg, [HR 1.56, 98.4% CI 1.22–2.00; *p* < 0.001]; MAP ≤ 55 mmHg, [HR 1.97, 98.4% CI 1.48–2.60; *p* < 0.001]) and 90-day mortality (MAP ≤ 65 mmHg, [HR 1.49, 98.4% CI 1.20–1.87; *p* < 0.001]; MAP ≤ 55 mmHg, [HR 1.78, 98.4% CI 1.38–2.31; *p* < 0.001]). POH of MAP ≤ 55 mmHg was also associated with greater risk of 7-day AKI stage II/III (HR 1.68, 98.4% CI 1.02–2.77; *p* = 0.013). No associations were observed between POH and 30-day readmissions, 30-day AMI, 30-day AIS and 7-day CRRT/dialysis for any MAP threshold. Detailed results for primary and secondary outcomes for all thresholds are shown in Fig. 3 and Additional file 1: Table S5, for *p* values. Unmeasured confounders required to reduce the odds ratio to 1.0 were evaluated using E-values for primary and secondary endpoints (Additional file 1: Table S6). E-values for significant endpoints ranged from 3.46 to 2.34 (MACCE ≤ 55 mmHg threshold to 90-day mortality ≤ 65 mmHg threshold).

### Association between POH and exploratory endpoints in the ICU

At any of the MAP thresholds investigated, no associations were found between POH and readmission to ICU after 72 h, ICU-free days in first 30 days and DAF of vasopressors (Additional file 1: Table S7 for exploratory outcomes).

## Discussion

This is the first study to examine the association between multiple postoperative hemodynamic thresholds in the absence of prior IOH (≤ 65 mmHg) with adverse clinical outcomes in the ICU setting. We demonstrate that POH at various hemodynamic thresholds (≤ 75, ≤ 65 and ≤ 55 mmHg) in the absence of IOH is associated with a larger absolute number of adverse clinical events as compared to patients with no POH exposure. Our findings underscore the importance of the immediate postoperative period (48 h) on patient outcomes among patients without intraoperative hypotension. Overall, the risk of MACCE and 30- and 90-day mortality progressively increased with decreasing MAP thresholds. For example, we saw an 11.1% increase in MACCE and 13.6% and 15.1% increase in 30- and 90-day mortality, respectively, in patients who had exposure to POH in the ICU, despite being stable in the intraoperative period.

The risk of experiencing 30-day MACCE, 30-day or 90-day mortality was significantly increased at blood pressure thresholds of ≤ 65 mmHg and ≤ 55 mmHg. The high incidence of 30-day MACCE (17.1%) in the overall population is likely driven by 30-day mortality (16.3%), as 30-day AMI (1.2%) and AIS (2.6%) had a relatively low incidence in our population. This would also explain the lack of significant demonstrable associations of POH in the ICU with AMI or AIS. Our mortality rate in this critically ill population is supported by other recent studies in surgical and non-surgical ICU populations, which report rates of mortality between 16 and 20% [1, 30–34]. In particular, our mortality rate is consistent with a large cohort analysis of ~ 50,000 patients across 65 ICUs in Sweden which reported a 30-day mortality rate of 17% [32]. Indeed, our study population had 36% of overall patients (across all blood pressure thresholds) classified with CCI ≥ 4, suggesting that these patients were suffering from a number of comorbid conditions prior to their non-cardiac surgery, which could have contributed to adverse events with an associated increased risk of mortality.

Khanna and colleagues reported that increasing amounts of hypotension at pressures previously regarded as normal were associated with myocardial injury, mortality and renal injury among surgical ICU patients; however, there was an interaction between IOH and critical care outcomes related to POH, making it difficult to evaluate the selective contribution of POH among ICU patients in the absence of IOH [8]. Therefore, our study builds upon Khanna and colleagues as it effectively evaluated the association of POH with adverse clinical events in postoperative critical care patients that did not experience intraoperative hypotension. Our observations that POH in the ICU with MAP ≤ 65 mmHg is associated with increased risk of MACCE as well as 30- and 90-day mortality suggest that POH in the critically ill is hazardous and blood pressure should be carefully, and preferably, continuously monitored using invasive and non-invasive methods as clinically indicated. Furthermore, this study evaluated several hemodynamic thresholds and demonstrated that POH, even at a MAP of 75 mmHg, though not statistically significant, results in an increased rate of adverse clinical events (MACCE, mortality measures and AKI/CRRT) compared to maintaining MAP above 75 mmHg. This finding is novel and opens room to question conventional wisdom of MAP goal 65 mmHg in ICU patients, as illustrated by a recent editorial [35]. Other randomized trials of blood pressure in the ICU have not found a difference in outcomes comparing different blood pressure targets, though these were septic patients and different from our novel postoperative ICU population that had a stable intraoperative course prior to ICU admission [36, 37]. Overall, our data suggest the importance of aggressive monitoring and early intervention to correct blood pressure in the ICU which may decrease mortality and adverse clinical events.

Our study has several limitations which must be considered, the first being that as this study was observational in nature, the data were subject to reporting bias/data entry errors. While we calculated E-values to measure uncontrolled confounding, we cannot exclude that it may not exist. Overall, we controlled for numerous confounders available in our dataset, however, if hypotension was simply an indicator of the degree of severity of the underlying illness, our estimated risks could be overestimations [1]. Second, since this was a retrospective study, we had no control over the treatments chosen for the hypotension events and the specific protocols within and across various hospitals. Third, while we made every attempt to minimize gaps between blood pressure readings (limiting gaps to one 2-h gap in-between readings), this presents a limitation (therefore time below thresholds as time-weighted averages was not modeled). One reason for this is that only nurse or provider validated blood pressure readings were entered into electronic records; therefore, we sacrificed blood pressure data granularity at the cost of possibly greater accuracy. Additionally, since MAP recordings were intermittent in nature, there was the potential for unstable MAP readings especially at higher MAP values which might have resulted in higher average times below the MAP thresholds. Fourth, our study population was focused on ICU patients; therefore, our results cannot be generalized to surgical patients discharged to the ward after the operative procedure. Fifth, AKI stage II/III was chosen as an outcome for this study because at the time a large volume of literature suggested that hospital mortality was associated with stage II/III renal dysfunction [24]. Although, we realize now that AKI stage I has similar long-term renal dysfunction when compared with stage II/III [38]. Sixth, our study design allows for a patient to have multiple non-cardiac procedures in our cohort as procedures > 30 days prior to the index visit could also be included in this study. Therefore, any hypotension experienced during or after that procedure could have resulted in unknown long-term effects. Finally, because we utilized an algorithm with a positive predictive value of 0.97 to help assign patients without a known care location to the ICU, 3% of patients could be misidentified.

## Conclusions

In conclusion, our analyses revealed a strong association between POH at several different hemodynamic thresholds in critical care patients with no hypotensive episodes during surgery (MAP > 65 mmHg), and adverse clinical outcomes in a multi-center retrospective cohort post-non-cardiac surgery. This study advances the field on POH among the critically ill as no study to date has evaluated the association among various POH thresholds in the absence of any prior hypotension with adverse clinical outcomes. The data here are hypothesis generating and should serve as a pilot for a larger interventional trial investigating the optimal blood pressure thresholds and need for improved hemodynamic monitoring for postoperative ICU patients, in order to improve short- and long-term outcomes of this vulnerable patient population.

## Supplementary information

**Additional file 1.** **Figure S1**. Initial attrition diagram leading into cohort selection for current study. **Method S1**. Brief description of the machine learning approach to identify patients discharged to the ICU for patients with an undocumented post-surgery care location. **Table S1**. Comorbidities and additional cohort patient characteristics. **Table S2**. International Classification of Diseases (ICD) codes. **Table S3**. List of antihypertensive drug classes. **Table S4**. Multivariate model results. **Table S5**. P-values of hazard ratios for primary and secondary outcomes. **Table S6**. E-values to assess the magnitude of an unobserved or unaccounted confounding effect for postoperative hypotension in intensive care unit setting. **Table S7**. Hazard/Incidence rate ratios and p-values for length-of-stay and use of intensive care unit-specific endpoints for intensive care unit patients with postoperative hypotension (exploratory outcomes).

## Data Availability

The data that support the findings of this study are available from Optum® (Eden Prairie, MN), but restrictions apply to the availability of these data, which were used under license for the current study, and so are not publicly available. Data are, however, available from the authors upon reasonable request and with permission of Optum®.

## Abbreviations

AIS: Acute ischemic stroke
AKI: Acute kidney injury
AMI: Acute myocardial infarction
BP: Blood pressure
CCI: Charlson comorbidity index
CRRT: Continuous renal replacement therapy
CI: Confidence interval
CPT: Current procedural terminology
DAF: Days alive and free
HR: Hazard ratio
ICD: International Classification of Diseases
ICU: Intensive care unit
IOH: Intraoperative hypotension
IRR: Incidence rate ratio
MACCE: Major adverse cardiac or cerebrovascular events
MAP: Mean arterial pressure
POH: Postoperative hypotension
SBP: Systolic blood pressure
SD: Standard deviation
SDHR: Sub-distribution hazard ratio
