# Supplementary material for: Postoperative hypotension in patients discharged to the intensive care unit after non-cardiac surgery is associated with adverse clinical outcomes
Source: Crit Care. 2020 Dec 7;24:682. doi: 10.1186/s13054-020-03412-5 (PMC7720547; doi:10.1186/s13054-020-03412-5)
Supplement: Supplementary file 1 — Additional file 1. Figure S1. Initial attrition diagram leading into cohort selection for current study. Method S1. Brief description of the machine learning approach to identify patients discharged to the ICU for patients with an undocumented post-surgery care location. Table S1. Comorbidities and additional cohort patient characteristics. Table S2. International Classification of Diseases (ICD) codes. Table S3. List of antihypertensive drug classes. Table S4. Multivariate model results. Table S5. P-values of hazard ratios for primary and secondary outcomes. Table S6. E-values to assess the magnitude of an unobserved or unaccounted confounding effect for postoperative hypotension in intensive care unit setting. Table S7. Hazard/Incidence rate ratios and p-values for length-of-stay and use of intensive care unit-specific endpoints for intensive care unit patients with postoperative hypotension (exploratory outcomes). [file 13054_2020_3412_MOESM1_ESM.pdf]

# Additional File 1


| Figure / Table | Figure/ Table Title | Page Number |
|---|---|---|
| Figure S1 | Initial attrition diagram leading into cohort selection for current study | 2 |
| Method S1 | Brief description of the machine learning approach to identify patients discharged to the ICU for patients with an undocumented post-surgery care location. | 3 |
| Table S1 | Comorbidities and additional cohort patient characteristics (laboratory values and surgical demographics) | 4 |
| Table S2 | International Classification of Diseases (ICD) codes | 6 |
| Table S3 | List of antihypertensive drug classes | 8 |
| Table S4 | Multivariate model results | 9 |
| Table S5 | P-values of hazard ratios for primary and secondary outcomes | 13 |
| Table S6 | E-values to assess the magnitude of an unobserved or unaccounted confounding effect for postoperative hypotension in intensive care unit setting | 14 |
| Table S7 | Impact of postoperative hypotension in the intensive care unit on length of stay and use of intensive care unit-specific endpoints (exploratory outcomes) | 15 |

**Figure S1. Initial attrition diagram leading into cohort selection for current study**

**621,482**: Non-cardiac and non-obstetric section surgical procedures from 2008-2017 with ≥10 minutes of MAP recordings at ≤ 5-minute intervals between readings, allowing for no more than two 5- to 10-minute gaps between MAP readings

**Surgeries excluded from the set:**

- **2,088:** Surgery types with count < 10 in cohort
- **194,721:** Patients with no touchpoint in the year before or no touchpoint or death in the year after
- **372:** Surgeries within ± 3 days of cardiac surgery
- **1,140:** No MAP reading in the 6 months prior to surgery
- **2,335:** No documented age or sex for patient
- **15,400:** Same day admission and discharge
- **11,206:** Missing MAP values during OR period
- **8,266**: Surgeries within 30 days of one another
- **17,132:** Patients with invalid dates of death

**368,222** surgeries in the initial cohort

Footnote: MAP: mean arterial pressure; OR: operating room.

**Method S1: Brief description of the machine learning approach to identify patients discharged to the ICU for patients with an undocumented post-surgery care location.** Originally utilized in: Khanna AK, Shaw AD, Stapelfeldt WH, Boero IJ, Chen Q, Stevens M, et al. Postoperative Hypotension is Associated with Adverse Clinical Outcomes in Patients Without Intraoperative Hypotension, After Non-Cardiac Surgery. (in submission). 2020.

- We utilized 19,216 intensive care unit and 111,572 ward patients for model development and validation.
- To build the model we included patients' demographics (age, race, gender, region, ethnicity, income, education), diagnosis and procedure codes, laboratory values, surgical related variables (length of surgery, type of surgery, time and day of surgery etc.,) and acute comorbidities (7-day sepsis/delirium/electrolyte disorder; 30-day acute kidney injury/acute myocardial infarction/acute ischemic stroke/prolonged mechanical ventilation/continuous renal replacement therapy/dialysis).
- Variables were standardized and missing values were imputed using median.
- The model was trained by using l1-based regularized logistic regression including both feature selection and coefficients penalization.
- Eight hundred and twenty-seven features were finally selected.
- In order to minimize false negatives (patients who discharged to the intensive care unit but were identified as ward by our algorithm), we selected the threshold as 0.79 with the positive predictive value as 0.97.

**Table S1: Comorbidities and additional cohort patient characteristics.** Laboratory values and surgical demographics for non-cardiac/non-obstetric surgery patients discharged to the ICU after surgery <u>without</u> intraoperative hypotension (MAP ≤65-mmHg)

| Patient Characteristics | Overall (n = 3,185) | POH MAP Threshold ≤55-mmHg (n = 654) | ≤65-mmHg (n = 1,688) | ≤75-mmHg (n = 2,674) | >75-mmHg (n = 511) |
|---|---|---|---|---|---|
| **Baseline Creatine before surgery (closest test in the six months before surgery)** | | | | | |
| ≤0.9 | 1,717 (53.9%) | 292 (44.7%) | 853 (50.5%) | 1,427 (53.4%) | 290 (56.8%) |
| 1.0 - 1.4 | 798 (25.1%) | 187 (28.6%) | 436 (25.8%) | 678 (25.4%) | 120 (23.5%) |
| 1.5 - 1.9 | 220 (6.9%) | 55 (8.4%) | 127 (7.5%) | 184 (6.9%) | 36 (7.1%) |
| ≥2.0 | 378 (11.9%) | 111 (17.0%) | 245 (14.5%) | 329 (12.3%) | 49 (9.6%) |
| unknown | 72 (2.3%) | 9 (1.4%) | 27 (1.6%) | 56 (2.1%) | 16 (3.1%) |
| **Baseline Hemoglobin before surgery (closest test in the six months before surgery)** | | | | | |
| ≤10.0 | 954 (30.0%) | 220 (33.6%) | 576 (34.1%) | 848 (31.7%) | 106 (20.7%) |
| 10.1 - 13.0 | 1,370 (43.0%) | 297 (45.4%) | 741 (43.6%) | 1,159 (43.3%) | 211 (41.3%) |
| 13.1 - 15.0 | 604 (19.0%) | 104 (15.9%) | 258 (15.3%) | 466 (17.4%) | 138 (27.0%) |
| ≥15.1 | 202 (6.3%) | 30 (4.6%) | 95 (5.6%) | 160 (6.0%) | 42 (8.2%) |
| unknown | 55 (1.7%) | 3 (0.5%) | 18 (1.1%) | 41 (1.5%) | 14 (2.7%) |
| **Surgery types (10 most common)*** | | | | | |
| Craniotomy | 799 (25%) | 63 (10%) | 264 (16%) | 600 (22%) | 199 (39%) |
| Thoracic surgery (non-cardiac, non-vascular) | 218 (7%) | 54 (8%) | 147 (9%) | 206 (8%) | 12 (2%) |
| Colon | 192 (6%) | 52 (8%) | 117 (7%) | 166 (6%) | 26 (5%) |
| Spinal fusion | 139 (4%) | 41 (6%) | 94 (6%) | 126 (5%) | 13 (3%) |
| Open reduction of fracture | 83 (3%) | 31 (5%) | 55 (3%) | 73 (3%) | 10 (2%) |
| Gallbladder | 70 (2%) | 21 (3%) | 36 (2%) | 61 (2%) | 9 (2%) |
| Limb amputation | 61 (2%) | 18 (3%) | 41 (2%) | 55 (2%) | 6 (1%) |
| Spinal fusion laminectomy | 55 (2%) | 15 (2%) | 37 (2%) | 48 (2%) | 7 (1%) |
| Hip prosthesis | 55 (2%) | 19 (3%) | 48 (3%) | 55 (2%) | 0 (0%) |
| Knee prothesis | 12 (0.4%) | 4 (1%) | 7 (0.4%) | 9 (0.3%) | 3 (1%) |
| Other | 1,501 (47%) | 336 (51%) | 842 (50%) | 1,275 (48%) | 226 (44%) |
| **Year of surgery** | | | | | |
| 2008-2011[#] | 248 (8%) | 65 (10%) | 146 (9%) | 205 (8%) | 43 (8%) |
| 2012-2013 | 639 (20%) | 142 (22%) | 364 (22%) | 552 (21%) | 87 (17%) |
| 2014-2015 | 1,135 (36%) | 235 (36%) | 590 (35%) | 941 (35%) | 194 (38%) |
| 2016-2017 | 1,163 (37%) | 212 (32%) | 588 (35%) | 976 (37%) | 187 (37%) |
| **Length of surgery** | | | | | |
| <1 hour | 2,558 (80%) | 527 (81%) | 1,366 (81%) | 2,158 (81%) | 400 (78%) |
| 1-2 hours | 511 (16%) | 99 (15%) | 264 (16%) | 423 (16%) | 88 (17%) |
| >2 hours | 116 (4%) | 28 (4%) | 58 (3%) | 93 (3%) | 23 (5%) |
| **Date of surgery** | | | | | |
| Weekend | 843 (26%) | 173 (26%) | 438 (26%) | 709 (27%) | 134 (26%) |
| Night | 576 (18%) | 145 (22%) | 326 (19%) | 489 (18%) | 87 (17%) |

(continued)

**Table S1: Comorbidities and additional cohort patient characteristics.** Laboratory values and surgical demographics for non-cardiac/non-obstetric surgery patients discharged to the ICU after surgery without intraoperative hypotension (MAP ≤65-mmHg) (continued)

| Patient Characteristics | Overall (n = 3,185) | POH MAP Threshold ≤55-mmHg (n = 654) | ≤65-mmHg (n = 1,688) | ≤75-mmHg (n = 2,674) | >75-mmHg (n = 511) |
|---|---|---|---|---|---|
| **Time dependent variables** | | | | | |
| Evidence of major bleeding | 740 (23%) | 97 (15%) | 319 (19%) | 601 (22%) | 139 (27%) |
| Vasopressor | 447 (14%) | 211 (32%) | 370 (22%) | 427 (16%) | 20 (4%) |
| Antihypertensives | 2,124 (67%) | 409 (63%) | 1,102 (65%) | 1,761 (66%) | 363 (71%) |

*Footnote: Due to rounding, categories will not always add to 100%. POH: postoperative hypotension*
**Surgery types were not used for model adjustment but were used to evaluate the cumulative incidence of postoperative hypotension for the top 10 surgeries, among patients discharged to the ICU for 48-hours post non-cardiac/non-obstetric surgery.*
*#Four years were combined due to small sample size in 2008 and 2009.*

**Table S2. International Classification of Diseases (ICD) codes**

| Condition | ICD 9 | ICD 10 | CPT |
|---|---|---|---|
| Prior myocardial infarction | 410, 412 | I21, I22, I252 | |
| Cerebrovascular accident | 430, 431, 433, 434, 436-438 | I60-I63, I6782, I69 | |
| Chronic obstructive pulmonary disease | 49, 500-505 | I278, I279, J684, J701, J703, J40-J47, J60-J67 | |
| Heart failure | 428 | I0981, I110, I130, I132, I50, I9713 | |
| Valvular heart disease | 424, 394-397 | I34-I39, I05-I07 | |
| Pulmonary circulatory disorder | 416 | I27 | |
| Peripheral vascular disease | 4439, 441, 7854, V434 | I70, I71, I73, I771, I790, I792, K551, K558, K559, Z958, Z959 | |
| Hypertension | 401, 4160 | I10-I13, I15, I16 | |
| Paralysis | 3441, 342 | G041, G114, G801, G802, G81-G83 | |
| Diabetes | 249, 250 | E08-E11, E13, E14 | |
| Hypothyroidism | 243, 244 | E03, E890 | |
| Renal disease | 582, 583, 585, 586, 588 | I12, I13, N03, N05, N17-N19, N25-N29, Z49, Z940, Z992 | |
| Liver disease | 570-573 | K70-K77 | |
| Lymphoma | 200-202 | C81-C86, C88 | |
| Solid tumor (local + meta) | 140-172, 174-198, 1990, 1991, 200-208 | C0, C1, C20-C26, C30-C34, C37-C41, C43, C45-C58, C60-C85, C88, C90-C97 | |
| Rheumatoid arthritis/collagen vascular disease | 3571, 710, 714 | M05, M06, M32, M34, M35 | |
| Coagulopathy | 286 | D65-D68 | |
| Obesity | 278 | E66 | |
| Anemia | 280-285 | D50-D53, D55-D59, D61-D64 | |
| Alcohol abuse | 291, 303, 3050 | F10, Z714 (excluding F1022) | |
| Drug abuse | 292, 304, 305 (excluding 3051, 3050, 30500, 30501, 30502, 30503, 3059, 30590, 30591, 30592, 30593) | F11-F19 (excluding F1111, F1121, F1211, F1221, F1311, F1321, F1411, F1421, F1511, F1521, F1611, F1621, F17210, F17211, F17213, F17218, F17219, F17200, F17201, F17203, F17208, F17209, F17221, F17291, F1811, F1821, F1911, F1921) | |
| Smoking | 3051, 3052 | F1720, F1721 | |
| Depression | 296, 311 | F32, F33, F34 | |
| Sleep apnea | 32721, 32723, 32727, 78057 | G4730, G4731, G4733, G4737, R0681 | |
| Dementia | 290 | F051, G311, F00, F01, F02, F03, G30 | |
| Supplemental oxygen use | V462 | Z9981 | |
| Dialysis | 39.95, 54.98 | 5A1D70Z, 5A1D80Z, 5A1D90Z, 5A1D00Z, 3E1M39Z | 90935, 90937, 90945, 90947, 4054F, 4055F |

(continued)

**Table S2. International Classification of Diseases (ICD) codes (continued)**

| Condition | ICD 9 | ICD 10 | CPT |
|---|---|---|---|
| Coronary artery bypass grafting | 36.10-36.16, 36.2 | 0210093, 02100A3, 02100J3, 02100K3, 02100Z3, 210493, 02104A3, 02104J3, 02104K3, 02104Z3, 021008W, 021009W, 02100AW, 02100JW, 02100KW, 021048W, 021049W, 02104AW, 02104JW, 02104KW, 021108W, 021109W, 02110AW, 02110JW, 02110KW, 021148W, 021149W, 02114AW, 02114JW, 02114KW, 021208W, 021209W, 02120AW, 02120JW, 02120KW, 021248W, 021249W, 02124AW, 02124JW, 02124KW, 021308W, 021309W, 02130AW, 02130JW, 02130KW, 021348W, 021349W, 02134AW, 02134JW, 02134KW, 210088, 210089, 021008C, 210098, 210099, 021009C, 02100A8, 02100A9, 02100AC, 02100J8, 02100J9, 02100JC, 02100K8, 02100K9, 02100KC, 02100Z8, 02100Z9, 02100ZC, 210488, 210489, 021048C, 210498, 210499, 021049C, 02104A8, 02104A9, 02104AC, 02104J8, 02104J9, 02104JC, 02104K8, 02104K9, 02104KC, 02104Z8, 02104Z9, 02104ZC, 211088, 211089, 021108C, 211098, 211099, 021109C, 02110A8, 02110A9, 02110AC, 02110J8, 02110J9, 02110JC, 02110K8, 02110K9, 02110KC, 02110Z8, 02110Z9, 02110ZC, 211488, 211489, 021148C, 211498, 211499, 021149C, 02114A8, 02114A9, 02114AC, 02114J8, 02114J9, 02114JC, 02114K8, 02114K9, 02114KC, 02114Z8, 02114Z9, 02114ZC, 021208C, 021209C, 02120AC, 02120JC, 02120KC, 02120ZC, 021248C, 021249C, 02124AC, 02124JC, 02124KC, 02124ZC, 021308C, 021309C, 02130AC, 02130JC, 02130KC, 02130ZC, 021348C, 021349C, 02134AC, 02134JC, 02134KC, 02134ZC | 33510, 33511, 33512, 33513, 33514, 33516, 33517, 33518, 33519, 33521, 33522, 33523, 33530, 33534, 33535, 33536 |
| Percutaneous coronary intervention | 00.66, 36.07 | 02703, 02704, 02713, 02714, 02723, 02724, 02733, 02734 | 92920, 92924, 92928, 92933, 92937, 92941, 92943 |
| Continuous renal replacement therapy | 39.95 | 5A1D90Z | 90935, 90937, 4054F |
| Acute myocardial infarction | 410 | I21, I22 | |
| Acute ischemic stroke | 43301, 43311, 43321, 43331, 43381, 43391, 43401, 43411, 43491, 436, 4371 | I63 | |
| Acute kidney injury | 584 | N17 | |
| Delirium | F05 | 29011, 2903, 2930, 2931, 30011, 308, 78009, 78039 | |
| Electrolyte disorders | 276 | E87 | |
| Sepsis/SIRS | 028, 77181, 9959 | A40, A41, P36, R65 | |
| Right side valve disease* | moderate or severe tricuspid regurgitation/insufficiency | | |
| Left side valve disease* | moderate or severe mitral regurgitation/insufficiency, aortic regurgitation/insufficiency, or aortic stenosis | | |

**Using physicians' notes to define the variables*

**Table S3**: **List of antihypertensive drug classes**

| List of hypertensive drug classes |
|---|
| Loop diuretics |
| Beta-adrenergic antagonists (beta blockers) w/o ISA |
| HMG & CoA reductase inhibitors (statins) |
| Anticoagulants; coumarin derivatives |
| Alpha- & beta-adrenergic antagonists |
| Angiotensin-converting enzyme (ACE) inhibitors |
| Thiazides & related agents |
| Angiotensin II receptor antagonists (ARB) |
| Calcium channel antagonists (calcium channel blockers) |

## Table S4: Multivariate model results

| Patient Characteristics | POH MAP Threshold ≤55-mmHg (n = 654) p-value | Hazard Ratio (98.4%CI) | ≤65-mmHg (n = 1,688) p-value | Hazard Ratio (98.4%CI) | ≤75-mmHg (n = 2,674) p-value | Hazard Ratio (98.4%CI) |
|---|---|---|---|---|---|---|
| **Gender** | | | | | | |
| Male | | | reference | | | |
| Female | 0.6275 | 1.05 (0.82, 1.35) | 0.4382 | 1.08 (0.84, 1.39) | 0.3946 | 1.09 (0.85, 1.41) |
| **Race** | | | | | | |
| Caucasian | | | reference | | | |
| Non-white | 0.141 | 0.83 (0.62, 1.12) | 0.1467 | 0.84 (0.62, 1.13) | 0.1331 | 0.83 (0.61, 1.12) |
| **Age** | | | | | | |
| < 65 | | | reference | | | |
| 65+ | 0.5534 | 1.07 (0.81, 1.42) | 0.4831 | 1.09 (0.82, 1.44) | 0.3209 | 1.12 (0.85, 1.49) |
| **Region** | | | | | | |
| West | | | reference | | | |
| Midwest | 0.1313 | 1.42 (0.81, 2.50) | 0.1159 | 1.45 (0.82, 2.57) | 0.1001 | 1.48 (0.83, 2.62) |
| North | 0.6247 | 1.22 (0.46, 3.22) | 0.5938 | 1.24 (0.48, 3.21) | 0.6228 | 1.21 (0.47, 3.15) |
| South | 0.7755 | 1.07 (0.60, 1.92) | 0.6776 | 1.11 (0.62, 1.99) | 0.6384 | 1.12 (0.62, 2.02) |
| Other/Unknown | 0.5018 | 0.78 (0.31, 1.92) | 0.4835 | 0.77 (0.32, 1.87) | 0.5317 | 0.80 (0.33, 1.92) |
| **Within 30 days before surgery** | | | | | | |
| Acute kidney injury | 0.048 | 1.31 (0.94, 1.82) | 0.0473 | 1.31 (1.02, 1.73) | 0.0194 | 1.38 (0.99, 1.92) |
| Dialysis | 0.403 | 1.24 (0.67, 2.29) | 0.4589 | 1.21 (0.65, 2.24) | 0.4308 | 1.23 (0.66, 2.29) |
| **Within 7 days before surgery** | | | | | | |
| Delirium | 0.797 | 0.96 (0.65, 1.42) | 0.6796 | 0.93 (0.63, 1.39) | 0.5825 | 0.91 (0.61, 1.36) |
| Electrolyte disorder | 0.0125 | 1.32 (1.01, 1.72) | 0.0094 | 1.33 (1.02, 1.73) | 0.0039 | 1.37 (1.05, 1.78) |
| Sepsis | <0.0001 | 1.98 (1.49, 2.62) | <0.0001 | 1.96 (1.48, 2.59) | <.0001 | 1.38 (0.99, 1.92) |
| **Admitted from** | | | | | | |
| Home | | | reference | | | |
| Inpatient | 0.4121 | 1.10 (0.83, 1.45) | 0.4782 | 1.09 (0.82, 1.44) | 0.4253 | 1.10 (0.83, 1.46) |
| Skilled nursing facility | 0.0895 | 1.36 (0.88, 2.12) | 0.0942 | 1.36 (0.87, 2.10) | 0.1303 | 1.32 (0.85, 2.06) |
| Unknown | <.0001 | 3.29 (1.69, 6.42) | <.0001 | 3.52 (1.83, 6.79) | <.0001 | 3.61 (1.86, 7.02) |

(continued)

**Table S4: Multivariate model results (continued)**

| Patient Characteristics | POH MAP Threshold | | | | | |
|---|---|---|---|---|---|---|
| | ≤55-mmHg (n = 654) | | ≤65-mmHg (n = 1,688) | | ≤75-mmHg (n = 2,674) | |
| | p-value | Hazard Ratio (98.4%CI) | p-value | Hazard Ratio (98.4%CI) | p-value | Hazard Ratio (98.4%CI) |
| **Procedures in the year before date of surgery** | | | | | | |
| Coronary artery bypass graft | 0.4486 | 1.42 (0.47, 4.32) | 0.7094 | 1.20 (0.37, 3.89) | 0.622 | 1.26 (0.41, 3.85) |
| Percutaneous coronary intervention | 0.1534 | 0.44 (0.11, 1.75) | 0.1491 | 0.46 (0.13, 1.68) | 0.1422 | 0.47 (0.13, 1.63) |
| **Antihypertensive** | | | | | | |
| Use of antihypertensives | 0.9997 | 1.00 (0.65, 1.55) | 0.9002 | 1.02 (0.66, 1.58) | 0.9601 | 0.99 (0.64, 1.53) |
| **Comorbidities** | | | | | | |
| Myocardial infarction | 0.2745 | 1.19 (0.81, 1.73) | 0.3729 | 1.15 (0.78, 1.70) | 0.3362 | 1.17 (0.79, 1.72) |
| Cerebrovascular accident | <.0001 | 1.93 (1.42, 2.62) | <.0001 | 1.94 (1.43, 2.63) | <.0001 | 1.95 (1.44, 2.64) |
| COPD | 0.1639 | 1.17 (0.89, 1.53) | 0.1044 | 1.20 (0.92, 1.57) | 0.0842 | 1.21 (0.93, 1.58) |
| Heart failure | 0.2974 | 1.14 (0.84, 1.56) | 0.3504 | 1.13 (0.82, 1.55) | 0.2608 | 1.16 (0.85, 1.58) |
| Pulmonary circulatory disorder | 0.0665 | 1.35 (0.91, 2.02) | 0.037 | 0.80 (0.58, 1.09) | 0.0262 | 1.44 (0.97, 2.15) |
| Peripheral vascular disease | 0.0887 | 0.80 (0.58, 1.10) | 0.0818 | 1.37 (0.98, 1.90) | 0.0729 | 0.79 (0.58, 1.08) |
| Hypertension | 0.0298 | 1.34 (0.97, 1.86) | 0.0223 | 1.15 (0.78, 1.70) | 0.0235 | 1.37 (0.98, 1.91) |
| Paralysis | 0.4698 | 1.12 (0.76, 1.66) | 0.3802 | 1.16 (0.89, 1.52) | 0.4911 | 1.12 (0.75, 1.66) |
| Diabetes | 0.1703 | 1.16 (0.89, 1.52) | 0.1801 | 1.00 (0.74, 1.36) | 0.1562 | 1.17 (0.90, 1.53) |
| Hypothyroidism | 0.8481 | 0.98 (0.72, 1.33) | 0.9919 | 0.75 (0.52, 1.07) | 0.9493 | 1.01 (0.74, 1.37) |
| Renal disease | 0.0419 | 0.74 (0.51, 1.06) | 0.0487 | 0.75 (0.52, 1.07) | 0.0284 | 0.72 (0.50, 1.03) |
| Liver disease | 0.1348 | 1.23 (0.88, 1.71) | 0.1674 | 1.21 (0.87, 1.69) | 0.1066 | 1.25 (0.89, 1.75) |
| Lymphoma | 0.4445 | 0.75 (0.31, 1.84) | 0.437 | 0.76 (0.32, 1.78) | 0.4478 | 0.75 (0.31, 1.85) |
| Solid tumor (local) | 0.0098 | 1.39 (1.02, 1.88) | 0.0046 | 1.43 (1.05, 1.93) | 0.0065 | 1.41 (1.04, 1.92) |
| RA/ collagen vascular disease | 0.9999 | 1.00 (0.59, 1.68) | 0.9328 | 0.98 (0.58, 1.65) | 0.8559 | 0.96 (0.56, 1.64) |
| Coagulopathy | 0.0041 | 1.51 (1.07, 2.14) | 0.0072 | 1.48 (1.04, 2.10) | 0.0118 | 1.45 (1.02, 2.06) |
| Obesity | 0.0013 | 0.65 (0.47, 0.90) | 0.0018 | 0.66 (0.47, 0.91) | 0.0027 | 0.67 (0.48, 0.92) |
| Anemia | 0.9339 | 0.99 (0.75, 1.30) | 0.9218 | 0.99 (0.75, 1.30) | 0.9111 | 1.01 (0.77, 1.34) |
| Alcohol abuse | 0.36 | 0.85 (0.56, 1.30) | 0.5294 | 0.90 (0.59, 1.36) | 0.397 | 0.86 (0.55, 1.33) |
| Drug abuse | 0.36 | 0.84 (0.54, 1.32) | 0.3888 | 0.85 (0.54, 1.33) | 0.4209 | 0.86 (0.55, 1.35) |
| Smoking | 0.2476 | 1.15 (0.86, 1.53) | 0.2666 | 1.14 (0.86, 1.52) | 0.2785 | 1.14 (0.85, 1.51) |

(continued)

## Table S4: Multivariate model results (continued)

| Patient Characteristics | POH MAP Threshold ≤55-mmHg (n = 654) p-value | ≤55-mmHg Hazard Ratio (98.4%CI) | ≤65-mmHg (n = 1,688) p-value | ≤65-mmHg Hazard Ratio (98.4%CI) | ≤75-mmHg (n = 2,674) p-value | ≤75-mmHg Hazard Ratio (98.4%CI) |
|---|---|---|---|---|---|---|
| **Comorbidities (continued)** | | | | | | |
| Depression | 0.394 | 0.90 (0.68, 1.20) | 0.467 | 0.92 (0.69, 1.22) | 0.3109 | 0.88 (0.66, 1.18) |
| Sleep apnea | 0.3649 | 0.87 (0.60, 1.26) | 0.3435 | 0.86 (0.60, 1.25) | 0.27 | 0.84 (0.58, 1.22) |
| Dementia | 0.0403 | 1.47 (0.93, 2.31) | 0.0338 | 1.49 (0.95, 2.35) | 0.0315 | 1.50 (0.95, 2.38) |
| Home oxygen | 0.818 | 1.05 (0.65, 1.69) | 0.7575 | 1.06 (0.66, 1.72) | 0.8185 | 1.05 (0.64, 1.71) |
| Right side valve disease | 0.0499 | 1.79 (0.87, 3.68) | 0.0391 | 1.83 (0.90, 3.69) | 0.0472 | 1.80 (0.88, 3.66) |
| Left side valve disease | 0.226 | 0.68 (0.31, 1.47) | 0.216 | 0.68 (0.31, 1.45) | 0.2236 | 0.68 (0.32, 1.46) |
| **Charlson Comorbidity Index (CCI)** | | | | | | |
| CCI 0-1 | | | reference | | | |
| CCI 2-3 | 0.2522 | 0.85 (0.60, 1.20) | 0.2469 | 0.85 (0.60, 1.19) | 0.3958 | 0.89 (0.63, 1.25) |
| CCI ≥4 | 0.7964 | 1.05 (0.69, 1.59) | 0.8472 | 1.03 (0.68, 1.56) | 0.7144 | 1.07 (0.70, 1.61) |
| **Income** | | | | | | |
| Quartile 1 | | | reference | | | |
| Quartile 2 | 0.0857 | 1.26 (0.91, 1.73) | 0.0863 | 1.25 (0.91, 1.72) | 0.1169 | 1.23 (0.89, 1.69) |
| Quartile 3 | 0.2446 | 1.19 (0.83, 1.71) | 0.2932 | 1.17 (0.82, 1.68) | 0.3488 | 1.15 (0.80, 1.66) |
| Quartile 4 | 0.0152 | 1.56 (1.00, 2.44) | 0.0198 | 1.54 (0.99, 2.41) | 0.0259 | 1.51 (0.97, 2.36) |
| Missing value | 0.0166 | 1.95 (1.03, 3.70) | 0.0043 | 2.01 (1.11, 3.62) | 0.0034 | 2.01 (1.13, 3.57) |
| **Baseline Creatine before surgery (closest test in the six months before surgery)** | | | | | | |
| ≤0.9 | | | reference | | | |
| 1.0 - 1.4 | 0.0671 | 1.27 (0.93, 1.75) | 0.0408 | 1.31 (0.95, 1.79) | 0.0557 | 1.28 (0.94, 1.76) |
| 1.5 - 1.9 | 0.0696 | 1.43 (0.89, 2.31) | 0.0523 | 1.48 (0.91, 2.39) | 0.0721 | 1.43 (0.89, 2.31) |
| ≥2.0 | 0.0193 | 1.56 (0.99, 2.46) | 0.0154 | 1.58 (1.00, 2.49) | 0.023 | 1.53 (0.97, 2.41) |
| unknown | 0.6845 | 1.17 (0.46, 2.97) | 0.6317 | 1.20 (0.47, 3.05) | 0.7386 | 1.14 (0.44, 2.93) |
| **Baseline Hemoglobin before surgery (closest test in the six months before surgery)** | | | | | | |
| ≤10.0 | | | reference | | | |
| 10.1 - 13.0 | 0.2757 | 0.88 (0.66, 1.17) | 0.4392 | 0.91 (0.68, 1.21) | 0.4682 | 0.92 (0.69, 1.22) |
| 13.1 - 15.0 | 0.343 | 0.86 (0.58, 1.26) | 0.5142 | 0.90 (0.61, 1.33) | 0.5917 | 0.92 (0.62, 1.36) |
| ≥15.1 | 0.047 | 1.49 (0.92, 2.40) | 0.0245 | 1.56 (0.97, 2.52) | 0.0221 | 1.58 (0.98, 2.55) |
| unknown | 0.6152 | 0.81 (0.30, 2.21) | 0.4049 | 0.70 (0.25, 1.95) | 0.3161 | 0.64 (0.22, 1.85) |

(continued)

## Table S4: Multivariate model results (continued)

| Patient Characteristics | POH MAP Threshold ≤55-mmHg (n = 654) p-value | Hazard Ratio (98.4%CI) | ≤65-mmHg (n = 1,688) p-value | Hazard Ratio (98.4%CI) | ≤75-mmHg (n = 2,674) p-value | Hazard Ratio (98.4%CI) |
|---|---|---|---|---|---|---|
| **Year of surgery** | | | | | | |
| 2008-2014 | | | reference | | | |
| 2015-2017 | 0.7548 | 1.03 (0.81, 1.31) | 0.6054 | 1.05 (0.83, 1.34) | 0.7622 | 1.03 (0.81, 1.31) |
| **Length of surgery** | | | | | | |
| Surgical duration | 0.5724 | 1.00 (1.00, 1.00) | 0.5581 | 1.00 (1.00, 1.00) | 0.7521 | 1.00 (1.00, 1.00) |
| **Date of surgery** | | | | | | |
| Weekend | 0.2152 | 0.87 (0.66, 1.14) | 0.2151 | 0.87 (0.66, 1.14) | 0.1562 | 0.85 (0.65, 1.12) |
| Night | 0.0674 | 1.25 (0.93, 1.67) | 0.0236 | 1.31 (0.98, 1.75) | 0.0124 | 1.35 (1.01, 1.80) |
| **Time dependent variables** | | | | | | |
| Evidence of major bleeding | <.0001 | 2.14 (1.60, 2.85) | <.0001 | 1.97 (1.48, 2.63) | <.0001 | 1.92 (1.44, 2.56) |
| Vasopressor | 0.0006 | 1.60 (1.15, 2.24) | <.0001 | 2.03 (1.39, 2.95) | <.0001 | 2.64 (1.65, 4.21) |
| Antihypertensives | 0.1949 | 1.15 (0.88, 1.51) | 0.9266 | 0.99 (0.77, 1.28) | 0.5941 | 0.94 (0.71, 1.24) |

**Table S5. P-values of hazard ratios for primary and secondary outcomes.** P-values for hazard ratios describing the association of POH with primary and secondary endpoints among patients discharged to the ICU without IOH (MAP ≤65-mmHg) (n=3,185).

| **Outcome** | **POH Threshold** | | |
|---|---|---|---|
| | ≤55-mmHg | ≤65-mmHg | ≤75-mmHg |
| | p-value | p-value | p-value |
| 30-day MACCE | **<0.001*** | **<0.001*** | 0.224 |
| 30-day mortality | **<0.001*** | **<0.001*** | 0.099 |
| 90-day mortality | **<0.001*** | **<0.001*** | 0.143 |
| 30-day AIS | 0.877 | 0.820 | 0.717 |
| 7-day AKI | **0.013*** | 0.065 | 0.280 |
| 30-day AMI | 0.154 | 0.060 | 0.978 |
| 30-day readmission | 0.574 | 0.696 | 0.715 |
| Dialysis | 0.366 | 0.235 | 0.230 |
| Sepsis | 0.080 | 0.352 | 0.476 |

*Footnote: * Significant after applying Bonferroni adjustment (p-value of ≤ 0.05/3 or 0.016). POH: postoperative hypotension; MAP: mean arterial pressure; MACCE: major adverse cardiovascular and cerebrovascular events; AIS: acute ischemic stroke; AKI: acute kidney injury; AMI: acute myocardial infarction*

**Table S6. E-values to assess the magnitude of an unobserved or unaccounted confounding effect for postoperative hypotension in intensive care unit setting.** E-values were calculated as a sensitivity analysis to determine unobserved or unaccounted confounding effects for POH at three thresholds among patients without IOH (MAP ≤65-mmHg) for primary and secondary endpoints. E-values represent the effect size required for an unmeasured confounding to reduce the observed effect to OR 1.0.

| Outcome | POH Threshold | | |
|---|---|---|---|
| | MAP ≤55-mmHg | MAP ≤65-mmHg | MAP ≤75-mmHg |
| | E-value | E-value | E-value |
| 30-day MACCE | 3.46 | 2.41 | 1.67 |
| 30-day mortality | 3.35 | 2.49 | 1.83 |
| 90-day mortality | 2.96 | 2.34 | 1.69 |
| 30-day AIS | 1.31 | 1.34 | 1.53 |
| 7-day AKI | 2.75 | 2.17 | 1.83 |
| 30-day AMI | 3.91 | 4.15 | 1.11 |
| 30-day readmission | 1.36 | 1.24 | 1.28 |
| Dialysis | 2.13 | 2.34 | 2.45 |

*Footnote: POH: postoperative hypotension; MAP: mean arterial pressure; MACCE: major adverse cardiovascular and cerebrovascular events; AIS: acute ischemic stroke; AKI: acute kidney injury; AMI: acute myocardial infarction.*

*E-values were calculated as previously described by VanderWeele and Ding (VanderWeele TJ, Ding P. Sensitivity Analysis in Observational Research: Introducing the E-Value. Annals of Internal Medicine. 2017;167(4):268. Specifically, following formula was used*___:___ ***E-value=HR+sqrt{HR×(HR-1)}*** *This formula applies to a risk ratio greater than 1; for a risk ratio less than 1, we first took the inverse of the observed risk ratio and then applied the same formula.*

**Table S7. Hazard/Incidence rate ratios and p-values for length-of-stay and use of intensive care unit-specific endpoints for intensive care unit patients with postoperative hypotension (exploratory outcomes).** Data shown for procedures (n=3,185) without preceding IOH (≤65-mmHg) at three absolute POH thresholds (≤55-, ≤65-, and ≤75-mmHg).

| Outcome | POH Threshold | | | | | |
|---|---|---|---|---|---|---|
| | MAP ≤55-mmHg | | MAP ≤65-mmHg | | MAP ≤75-mmHg | |
| | Hazard Ratio (98.4%CI) | p-value | Hazard Ratio (98.4%CI) | p-value | Hazard Ratio (98.4%CI) | p-value |
| Readmission to ICU after 72 hours | 1.03 (0.36, 2.97) | 0.947 | 0.61 (0.27, 1.42) | 0.159 | 0.58 (0.21, 1.62) | 0.200 |
| | Incidence Rate Ratio (98.4%CI) | p-value | Incidence Rate Ratio (98.4%CI) | p-value | Incidence Rate Ratio (98.4%CI) | p-value |
| ICU free days in first 30-days | 0.92 (0.84, 1.01) | 0.026 | 0.95 (0.88, 1.01) | 0.056 | 0.98 (0.89, 1.08) | 0.623 |
| DAF of vasopressors | 0.98 (0.95, 1.00) | 0.020 | 0.98 (0.96, 1.00) | 0.063 | 0.99 (0.97, 1.02) | 0.647 |

*Footnote: * Significant after applying Bonferroni adjustment (p-value of ≤ 0.05/3 or 0.016). POH: postoperative hypotension; MAP: mean arterial pressure; ICU: intensive care unit; DAF: days alive and free.*
